# An RSSI Classification and Tracing Algorithm to Improve Trilateration-Based Positioning

**DOI:** 10.3390/s20154244

**Published:** 2020-07-30

**Authors:** Yong Shi, Wenzhong Shi, Xintao Liu, Xianjian Xiao

**Affiliations:** 1 School of Computer Information and Engineering, Changzhou Institute of Technology, Changzhou 21300, China; shiy@czu.cn (Y.S.); xiaoxj@czu.cn (X.X.); 2 Department of Land Surveying and Geo-Informatics, The Hong Kong Polytechnic University, Hong Kong; xintao.liu@polyu.edu.hk

**Keywords:** trilateral indoor positioning, RSSI filter, RSSI classification, stability, accuracy

## Abstract

Received signal strength indicator (RSSI)-based positioning is suitable for large-scale applications due to its advantages of low cost and high accuracy. However, it suffers from low stability because RSSI is easily blocked and easily interfered with by objects and environmental effects. Therefore, this paper proposed a tri-partition RSSI classification and its tracing algorithm as an RSSI filter. The proposed filter shows an available feature, where small test RSSI samples gain a low deviation of less than 1 dBm from a large RSSI sample collected about 10 min, and the sub-classification RSSIs conform to normal distribution when the minimum sample count is greater than 20. The proposed filter also offers several advantages compared to the mean filter, including lower variance range with an overall range of around 1 dBm, 25.9% decreased sample variance, and 65% probability of mitigating RSSI left-skewness. We experimentally confirmed the proposed filter worked in the path-loss exponent fitting and location computing, and a 4.45-fold improvement in positioning stability based on the sample standard variance, and positioning accuracy improved by 20.5% with an overall error of less than 1.46 m.

## 1. Introduction

With widespread Wi-Fi, Bluetooth (iBeacon), and smart mobile terminal deployment, received signal strength indicator (RSSI)-based indoor positioning technology has attracted research attention due to its advantages of low complexity, low cost and high accuracy [1,2]. However, the received signal strength (RSS) is easily blocked and easily interfered with by objects and environmental effects. These influences usually increase RSSI variance; thus, RSSIs vary sharply over time even when the actual signal strength remains constant. The variation reduces accuracy and stability for an RSSI-based indoor positioning system (IPS) [3,4,5]. Many assisted and combined technologies were proposed to achieve higher accuracy, e.g., pedestrian dead reckoning (PDR), computer vision, space-scenario, and artificial intelligence techniques [1,4]. However, fundamentally, dealing with RSSI is a crucial step during the whole IPS process.

RSSI is environment-dependent. Therefore, it is significant to filter the raw RSSIs before substituting them into the positioning process. Many RSSI purification technologies such as the Gaussian filter [6], Kalman filter [7], and particle filter [8,9] are typically designed to mitigate either the linear or non-linear noise through smoothing. Still, they may not effectively deal with the ever-changing dynamics of the indoor environment [10] and have left-skewed distributions [11]. The mean filter [12] is widely accepted because it has similar accuracy and anti-interference performance, but has less burden in filtering computation [13,14]. Besides smoothing, RSSI screening is another effective filtering method, e.g., by selecting the max N RSSIs (N = 13 is optimal) [13], and the least variance RSSIs over time [15]. Importantly, RSSI classification is also an effective filtering method, especially in combination with clustering algorithms for RSSI filtering and singular RSSI tracing [16,17].

RSSI-based IPS is generally divided into two categories: trilateration-based IPS and fingerprint-based IPS [10,18,19]. The fingerprint-based IPS [20,21,22] gets more concern in terms of the number of references retrieved, where the number is 1,910,000 vs. 30,000 from Google Scholar. It also gains positive effect by using unsupervised machine-learning algorithms to reduce the data dimensionality, and fingerprint matching calculations required [23]. Meanwhile, the trilateration-based positioning system is widely applied in outdoor environments [24,25]. However, it faces challenges in indoor environments, achieving 3–5 m accuracy without assistive technology and device, and lower stability [3]. The trilateration-based IPS positioning is easy to understand and easy to construct. Usually, RSSI-based trilateration IPS is undertaken in two main steps: distance mapping and position computation [10]. The distance between the unknown position sensor and the known position sensor anchor is obtained by some RSSI propagation model [26]; its accuracy depends on signal transmission anchor, path-loss exponent in the RSSI propagation model, and RSSI sampling, etc. [10,13]. Based on this, the unknown location is obtained by trilateration methods such as least square and maximum likelihood [4,10].

This paper will propose a new filtering technique to play a role in the propagation parameter fitting and the RSSI sampling purification, to improve RSSI-based IPS accuracy and stability, particularly for trilateration-based positioning, considering that accuracy and stability are equally important. The overall accuracy may diminish due to energy consumption [27,28], and stability may significantly influence the user experience and application promotion.

The main contributions of this study are as follows:

(1) We propose a tri-partition classification of RSSIs, considering that the interference effects are finite and unambiguous, increasing and decreasing while the interference sources are multiple and fuzzy. And we also propose a clustering algorithm to trace the tri-partition classification quantitatively and seek the partition distribution centers, helping to reveal the interference and judge whether the sun-classification conforms to normal distribution.
(2) We take the proposed algorithm as an RSSI filter and discuss its work mechanism. And we infer that it is feasible by analyzing the features in terms of sample count and deviation and advantages compared to the mean filter.
(3) We verify that the proposed filter works in path-loss exponent fitting and location computing, and analyze the improvement of IPS it yields.

The remainder of this paper is arranged as follows. In Section 2, we review RSSI-based trilateration positioning technologies and RSSI filters. We detail the proposed RSSI filter based on an RSSI classification and tracing algorithm in Section 3. In Section 4, we analyze the filtering performance, including features adapted to real-time IPS and advantages over the mean filter. Section 5 introduces a test to examine the positioning performance using the proposed filter and compares its performance with the mean filter. Finally, in Section 6, we summarize and conclude the paper.

## 2. Literature Review

### 2.1. Received Signal Strength Indicator (RSSI)-Based Trilateration Indoor Positioning System (IPS)

Trilateration-based positioning technology is easily understood and widely used in the positioning of pedestrians and robots and things [1,2,3]. In the method we should know the position of the anchors (reference nodes) as (*x*_1_, *y*_1_), (*x*_2_, *y*_2_), …, (*x*_n_, *y*_n_), and their distances from the target node, which is calculated by the RSSI-distance mapping, *d*_1_, *d*_2_, …, *d*_3_. If we assume the target node’s coordinates as (*x*, *y*), then the essential geometric functions as follows. When the anchors are more than 4, the least square and maximum likelihood [4,10] are used to calculate the target node’s optimal coordinates.

(1) $$\{\begin{array}{c} {\left(x_{1}-x\right)}^{2}+{\left(y_{1}-y\right)}^{2}=d_{1}{}^{2}\\ {\left(x_{2}-x\right)}^{2}+{\left(y_{2}-y\right)}^{2}=d_{2}{}^{2}\\ \ldots \\ {\left(x_{n}-x\right)}^{2}+{\left(y_{n}-y\right)}^{2}=d_{n}{}^{2} \end{array}$$

The positioning complexity is partly to know the anchor points’ location in advance, especially when anchors change and a lot of update work required. The difficulty is that RSSI-based trilateration techniques depend on an accurate estimation of distance by RSSI-distance mapping. RSSI is a function of distance and is generally affected by the environment and any changes therein. Usually, researchers use the following simplified propagation model to measure RSSI (Formula 1) and map RSSI to distance (Formula 2) [26,29,30]:

(2) ρ=α−10βlog(d)

(3) $$d=10^{\left(\left(\rho -\alpha \right)/\left(10*\beta \right)\right)}$$

where *d* is the distance from the current position to some beacon, ρ is the RSSI at the current position, *α* is the RSSI at some referenced distance (usually 1 m), *β* is the path-loss exponent, and the parameters *α* and *β* are obtained to adapt to different sensors and environments; the path-loss exponent *β* generally has a value in the range of 1.6–1.8 in an indoor environment [26,31,32]. More importantly, it is necessary to fit the path-loss exponent according to the actual environment in which RSSIs are collected [33,34]. These parameters should be calculated again when a target node moves across the boundary of two different environments [34], even be constantly updated if necessary [35]. Furthermore, the piecewise fitting and min–max method are proposed for local adaption to the real environment, 4 m and 8 m are the breakpoints, and the curvatures of different sections are noticeably different [15,34,36].

Accuracy is the most crucial performance metric of the positioning system. It is related to devices and its effective coverage. Wi-Fi and iBeacon belong to high-frequency signals, but the RSSI performance received is also different due to the difference in transmitting power and antenna angle. For example, iBeacon, particularly the loss of packets, is serious for the interval beyond 10 m [10,15,36]. The deployment density is also computing- and maintenance-cost related. When the anchors’ density is 0.27 nodes/m^2^, localization estimation error can be decreased to 1.5–2 m [37]. However, increasing the number of anchor nodes does not result in higher average accuracy, and with more than 50 anchors, the average accuracy declines [26]. Meanwhile, paper [38] proposes a sensor deployment method based on wireless sensor network topology optimization, and [39] suggests a novel technique related to pedestrian density, gaining an accuracy of 1.8–3.9 m.

Considering the overall accuracy may diminish due to energy consumption [27,28], that accuracy and stability are equally important, and stability may significantly influence the user experience and application promotion. This paper’s core contribution is to adopt the same positioning method without increasing the calculation amount of positioning, which reflects the function advantages of proposed classification and filtering in this paper.

### 2.2. RSSI Filtering Technologies

Raw RSSIs measurement is related to the parameter fitting and distance mapping process, and play a decisive role for IPS performance [33]. Compared with the mean filter [11], Kalman filter [7], particle filter [8,9], least-squares estimator [40,41], and maximum-likelihood estimator [42] have advantages in terms of accuracy but are computationally expensive. They use a moment before estimation and the current observations to update the state variables’ estimate, while the mean filter just takes the average. Thus, the mean filter is widely recommended because it has similar accuracy and anti-interference performance [11]. Considering their relatively small computational overheads and the fact they can be used in a real-time context, the rolling mean filter, exponential moving mean filer, and moving median filter have been discussed [12]. However, these filters are typically intended to mitigate these influences by smoothing and could suffer from left-skewed distributions caused by RSSI multipath propagation [15].

RSSI screening is another effective filtering method based on analyzing the spatial resolution of the signal strength and RSSI signal characteristics under different scenarios. Gaussian filter [6] selects the high RSSI probability RSSIs and takes the average value as filter results, lowers the influence of the small probability and interference over the measurement. An algorithm using the maximum RSSI average has been proposed and suggests that N = 13 is optimal [13]. Relative to selecting the maximum, [15] selected the least variance RSSIs over time, arguing that the normal variances are not dramatic. Based on dichotomy, Study [16] and [17] propose an RSSI classification to distinguish singular RSSIs from normal path-loss RSSIs. Paper [17] proposes a k-means clustering algorithm tracing the rating.

Applying artificial intelligence (including statistical inference methods) for RSSI processing is a new trend, e.g., using a k-means clustering algorithm for singular RSS tracing [22] and filtering, and RSSI fingerprint matching [23,24]. Moreover, support vector machine (SVM) [43], artificial neural network (ANN) [44,45] and deep learning [46] have been proposed to aid RSSI purification and high positioning accuracy. However, in indoor environments, all of the above algorithms still face challenges of spatial ambiguity, RSSI instability, and RSSI’s short collecting time per location [47,48].

This paper will propose a three-classification and tracking method to explore its distribution center instead of the mean center based on the classification idea and unsupervised learning algorithm. More importantly, we will discuss the filtering performance under the conditions of small samples and small sampling time.

## 3. RSSI Classification and Tracing

### 3.1. Tri-Partition RSSI Classification

**Figure 1:**
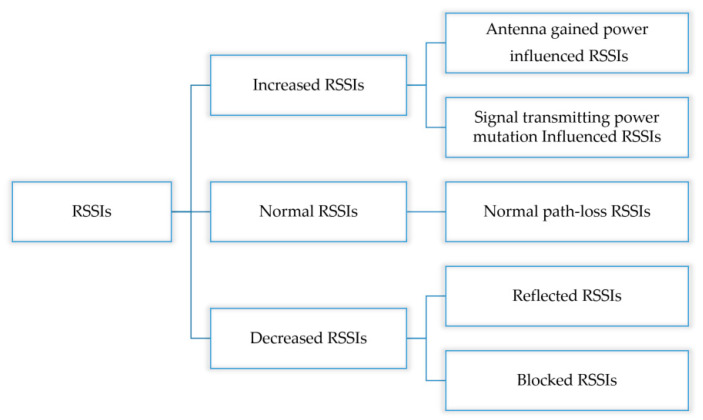
Received signal strength indicator (RSSI) classification based on the view of influence effects.

Environmental conditions, scenario changes, anchor deployment, transmission power, and interferences between anchor nodes can affect the RSSI values. Furthermore, it is challenging to determine antenna gains [10]. However, the actual effects can be summarized as increasing and decreasing, so unlike the dichotomy, we propose a tri-partition of RSSIs, as shown in Figure 1. We classify RSSI samples into three collections:

1. The decreased collection (DC) represents singular weakened RSSIs such as blocked and reflected signals.
2. The normal collection (NC) represents normal path-loss and fading RSSIs.
3. The increased collection (IC) represents singular enhanced RSSIs caused by transmitting equipment such as antenna gained power or transmitting power mutation.

The tri-partition can help with the quantitative analysis of the RSSI. Tracing the item count of sub-classification can reveal that the subsequent positioning is dependable or not, where NC RSSIs do not conform to normal distribution means more significant errors. Thus, it helps select different path-loss parameters to adapt to the environment.

### 3.2. RSSI Tracing Algorithm

We propose an RSSI tracing algorithm based on k-means clustering to determine the partition and its distribution center. The proposed algorithm considers the RSSI sample (R) to be a one-dimensional collection. It uses the absolute value of RSSI and sub-classification center subtraction as the clustering factor, takes the maximum RSSI as the initial center of the IC, the minimum RSSI as the initial center of the DC, the mean RSSI as the initial center of the NC, and then defines the assistant function MIN(R) to obtain the minimum RSSI from R, the function MAX(R) to obtain the maximum RSSI from R, and the function AVERAGE(R) to obtain the mean value from R. The algorithm steps are as follows Algorithm 1:
**Algorithm 1.** Sub-Classification Tracing.Input: R = {RSSI_1_, RSSI_2_, …, RSSI_n_}//the collected RSSI samples Output: NC, IC, DCDefine://initial the center of IC, NC, and DC    IV = MAX(R), NV = AVERAGE(R), and DV = MIN(R)    IC_T_ = ϕ, NC_T_ = ϕ, and DC_T_ = ϕ  //temporal collections    TD = ID = ND = DD = 0.0    //temporal values. Classify: For each (RSSI_i_ ∈ R){ //calculate its distance to IV, NV, and DV ID = abs (IV- RSSI_i_)       ND = abs (NV- RSSI_i_)       DD = abs (DV- RSSI_i_)       //select the minimum distance       TD = MIN ({ID, ND, DD})        //add RSSIi to sub-classification       If (TD= =ID)       IC = IC + { RSSI_i_ }       Else if (TD= =ND)    NC = NC+{ RSSI_i_ }    Else    DC = DC + { RSSI_i_ }    } Judge:   //judge the change of sub-classification   If (IC_T_ = = NC_T_ = = DC_T_ = = ϕ){       IC_T_ = IC       NC_T_ = NC       DC_T_ = DC       } GOTO (Reset) //if convergence, exit   If (IC_T_ = = IC and NC_T_ = = NC and DC_T_ = = DC)   GOTO (Exit)   Else {       IC_T_ = IC       NC_T_ = NC       DC_T_ = DC       } Reset: //reset the sub-classification center IV = AVERAGE (IC)    NV = AVERAGE (NC)    DV = AVERAGE (DC)    GOTO (Classify) Exit:  //deal with ϕ   If (NC = = ϕ) {    TD = (ID + DD)/2    NC+ = {TD}    } EXIT(-)
where NC equals ϕ in the Algorithm (1) means that sample R has a polarization distribution, and partition NC is affected by substantial RSSI deviation from reality. When we set the mean value as a new RSSI, the whole count will increase by one each time.

### 3.3. Apply to Trilateration-Based Positioning

**Figure 2:**
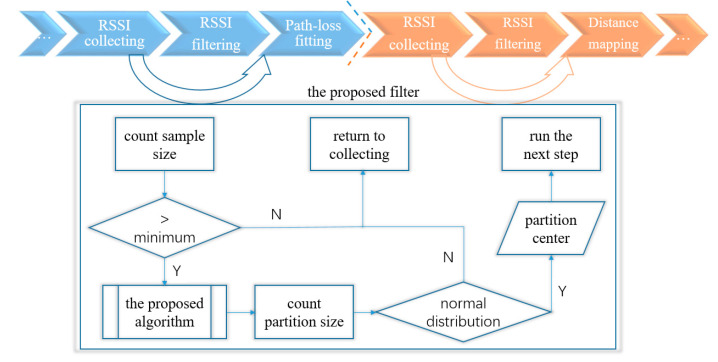
Application mechanism of the proposed filter for the indoor positioning system (IPS).

Trilateration-based IPS includes many steps [10,13], here we divide these steps into two stages: offline and online. The offline stage aims to adapt the path-loss exponent, including anchor deployment, RSSI collecting, raw RSSI measurement, and path-loss exponent fitting (may fit as needed during the online stage [15,34]). The online stage aims to optimize positioning, including real-time RSSI collecting, raw RSSI measurement, distance mapping, coordinate calculation, and positioning optimization and correction. This paper adopts the proposed tracing algorithm as an RSSI filter to re-establish the raw RSSI measurement for the path-loss exponent fitting (offline) and distance calculating (online) steps, as shown in Figure 2.

Figure 2 shows the application mechanism that focuses on the sample count and the partition item count. The minimum sample count is related to real-time positioning, as RSSI’s shortest collecting time per location usually less than 1 s. The partition item size is related to influences when the tri-partition counts conform to a normal distribution, indicating that the filtering result is reliable. For the filtering process, the partition center is the filtering result. Next, we will discuss the minimum sample and performance to reveal the availability of the proposed filter.

## 4. Feasibility and Performance as an RSSI Filter

To examine the proposed filter’s feasibility and performance advantages, we collected a large RSSI sample (named Sample ALL) over approximately 10 min using iBeacon as the signal sender. And we defined six type sample groups, named Sample X (X = 10, 20, 30, 40, 50, 60). Each sample group had 10 sample arrays, and each array had the same count RSSIs. The sample array data structure is as follows:

Test Sample = {Array_i,j_, I = 10, 20, …, 60, j = 1, 2, …, 10} (4)

where i represents different groups, and j represents different arrays in the current group. For example, Array_30,1_ represents that the collection is in group 30, and the collection’s item count is 30.

**Figure 3:**
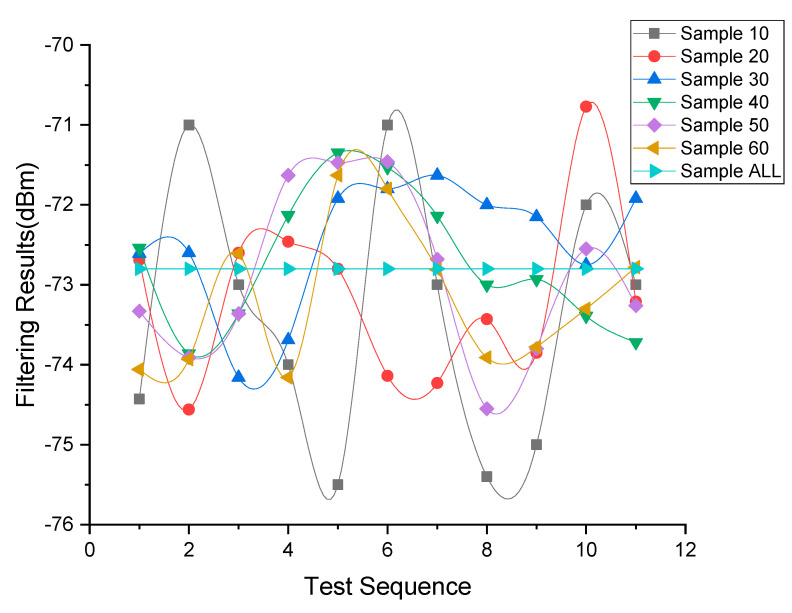
Filtering results of each test sample in sample group and the sample ALL.

In preparation, we cut the sample ALL into each test sample sequentially and continuously according to the sample type, and then filter each test sample and the sample ALL. Figure 3 shows the filtering results, where each node represents a filtering result of some Test Sample. For each type of sample, ten filtering tests are conducted in sequence, with a total of 60 filtering times. Figure 3 also shows Sample 10 has the max deviation from the Sample ALL, and with the sample count is larger than 20, the gap range gradually stabilized.

### 4.1. Features of the Proposed Filter

#### 4.1.1. Low Deviation

RSSI fluctuation is an inherent issue for wireless signals, hence reducing the variance range for each sample count is essential to improve the accuracy and stability [49,50]. Using the sample ALL as the comparative standard, we measured the deviation and variance of each test sample, which is as shown in Table 1. It summarizes the deviations from all samples, and together with Figure 3 shows that the proposed filter achieves lower variance. Sample 10 and Sample 20 have the highest variation with a maximum deviation range of less than 2.5 dBm and the mean deviation range of less than 1.44 dBm. When the test sample count was greater than 20, the maximum deviation range was less than 1.64 dBm, and the mean variation was below 0.92 dBm. As experiments have confirmed that a change of 1 dBm represents 0.08 m at some distance [13], therefore, the filter obtained a significantly lower fluctuation, and the test sample gains a similar performance to the sample. ALL, especially when the sample size is more significant than 20.

#### 4.1.2. Minimum Sample Count

While RSSIs are Gaussian distribution and random, the time of sampling can affect the collected RSSI count and positioning quality [10,51]. Most current IPSs and RSSI analyses take a long-time interval for sample collecting, such as 1–3 or 3–6 min, but it is a gap from second-level real-time requirements.

**Figure 4:**
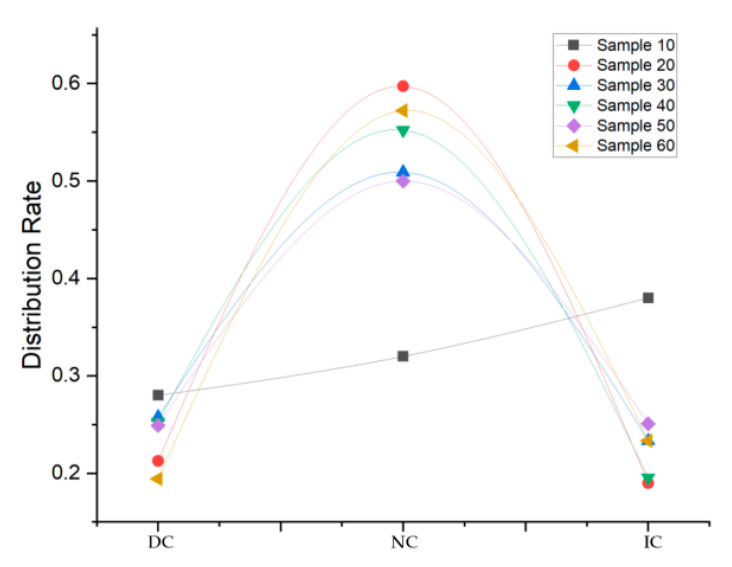
Distribution of tri-partition for sample groups.

By counting the items of each sample, as shown in Table 2, the Sample 10 had an item count of 103 when the expected count was 100, and sample 20 s item count was 201 when the expected count was 200, indicating that the NC in Sample 10 and Sample 20 generated ϕ during filtering. Further calculating the sub-classification distribution rate, as shown in Figure 4, the tri-partition collection items conform to the normal distribution when the sample count is over 20. Therefore, the proposed filter obtained a minimum sample count of 20 suitable for the positioning process.

In summary, the proposed filter has such features; the small test sample gains a similar performance to the larger sample ALL, and the sun-classification conforms to normal distribution when the sample count is larger than 20. Therefore, the proposed filter adapts to real-time positioning, for it has a lower deviation, and the minimum sample count is 20.

### 4.2. Advantages over the Mean Filter

**Figure 5:**
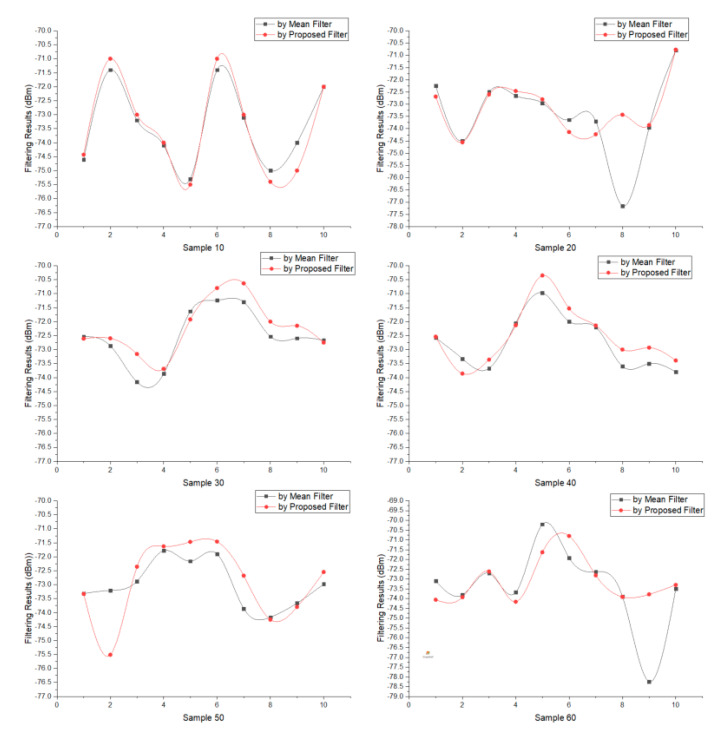
Filtering performance comparisons of each type sample by the proposed filter and the mean filter, respectively.

Using the same RSSI test sample from the upper section, Figure 5 shows the filtering results comparison by the proposed filter and the mean filter, respectively, where each node represents a filtering result of some test sample. For each type of sample, 10 filtering tests are conducted in sequence, with a total of 120 filtering computations. Based on the filtering performance, we discuss the advantages associated with reductions in variance below.

#### 4.2.1. Reducing Variance over Time

The comparisons use variance range and sample variance (SV, calculated using the sample standard deviation function in EXCEL) to measure the proposed and mean filters’ performance. As Figure 5 shows the filtering result, Table 3 shows the variance comparisons in detail. The proposed filter achieves a smaller maximum variance range than the mean filter, and a lower SV when the sample count is greater than 10.

Overall, the average maximum variance for the proposed filter was 2.31 dBm, while for the mean filter, this was 2.97 dBm. The average minimum deviation for the proposed filter was 0.37 dBm, while the mean filter was 0.39 dBm. The average variance for the proposed filter was 1.08 dBm, while the mean filter was 1.34 dBm, and the average SV for the proposed filter was 1.38 dBm, while the mean filter was 1.73 dBm. Thus, the proposed filter resulted in a lower and more stable variance.

#### 4.2.2. Reducing Left-Skewness

Traditional RSSI filtering commonly produces left-skewed distributions [11,26,50], i.e., the returned value is lower than the actual value. Table 4 shows that the proposed filter mitigates this problem, achieving a 65% superior performance in terms of skewness reduction. The left 35% which did not mitigate skewness; they had an average of 96.7% deviation at less than one dBm and an average of 78.9% deviation at less than 0.5 dBm, so the proposed filter successfully reduced skewness.

In summary, the proposed filter shows feasibility because it has significant low variance and a minimum sample of 20 properties and mitigating advantages over the mean filter which is vastly used.

## 5. Positioning Improvement

### 5.1. Experiment Design

**Figure 6:**
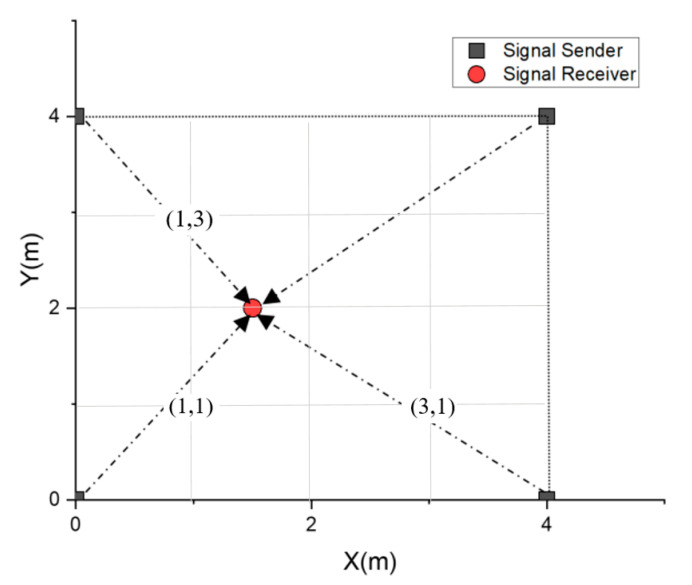
Testbed diagram and selected test points.

Figure 6 shows the 4 × 4 m testbed used to investigate the proposed filter’s positioning performance. The experiment selects (1,1), (1,3), and (3,3) as test points, deploys four iBeacon (SEEKCY s1u) sensors as RSS senders at the grid corners and selects an android mobile phone (MI max2) as the RSS receiver. To seek reliable positioning performance under real-time, we set the minimum sample count of 30 each time. The experimental steps are as follows.

1. Fit RSSI propagation model factors,
  - collect RSSI samples at each fixed distance (1 m, 2 m, …, 10 m),
  - filter each sample using the proposed and mean filters, and
  - fit the propagation model according to Formula (2).
2. For each positioning process,
  - filter RSSI samples using the proposed and mean filters,
  - perform traditional trilateral positioning, and
  - compare the positioning results.
3. Repeat step (2) for the next test process.

### 5.2. Experiment Results

#### 5.2.1. Propagation Factors

Using MATLAB Fitting Tools and a similar fitting process in paper [14,15], Table 5 shows the that *α* and β are distinctly different. It is foreseeable that the distance mapping process will still make a difference using the factors.

The *α* is as RSSI sample filtering result at 1 m, and the b is as the fitting result of collected RSSI samples at each fixed distance from 1 m to 10 m, by the proposed filter and the mean filter. Considering studies have confirmed that many packets may mass lose when the measured distance exceeds 10 m [36,52].

#### 5.2.2. Positioning Results

**Figure 7:**
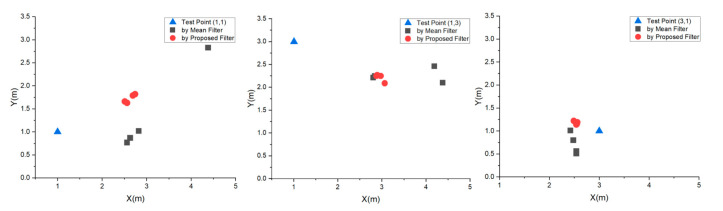
Positioning results distributions at point (1,1), point (1,3) and point (3,1).

Table 6 shows a total of 24 positioning coordinates. Figure 7 shows the positioning results and the relative positions of these test points. The proposed filter achieves a significantly compact location distribution at each test point, whereas the mean filter has a relatively loose distribution, with point (3,1) exhibiting the highest accuracy.

### 5.3. Improvement Analysis

#### 5.3.1. Accuracy

**Figure 8:**
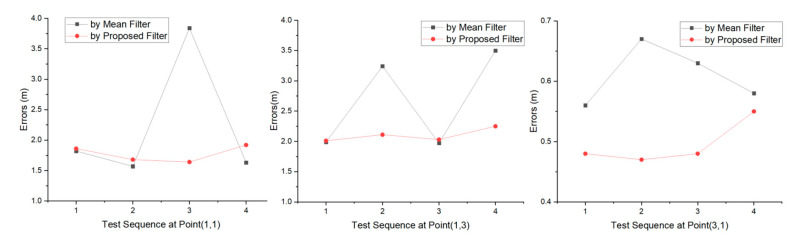
Positioning error comparison with the proposed filter and the mean filter at point (1,1), point (1,3) and point (3,1).

Figure 8 shows the calculated positioning errors in sequence. Based on the accuracy changes at each test point, errors by the proposed filter change little, while the errors by the mean filter change significantly. Unfortunately, despite the proposed filter gains in the overall improvement, it does not improve every positioning, and about 58% have higher accuracy compared to the mean filter.

In detail, Table 7 shows the comparison of the proposed filter and the mean filter in accuracy promotion. For each test point, the proposed filter achieves smaller maximum and average errors than the mean filter, and the positioning accuracy is improved by approximately 20% for each test point. The overall average error is 1.46 and 1.84 m for the proposed and mean filters, respectively, and the overall average accuracy improvement is 20.5%. Still, it can be concluded that even if the error by the proposed filter is larger, the difference between the errors by the mean filter is still small, this phenomenon is consistent with the mitigation performance analysis in chapter 4.

#### 5.3.2. Stability

Using sample standard deviation (SSD) to measure positioning stability, the SSD will be calculated by the following formula:

(5)  SSD=STDEV(R)/AVERAGE(R)

where *STDEV* (–) is the sample standard deviation function in EXCEL, and *AVERAGE* (–) is the mean function in EXCEL; *R* is the positioning error of each test point.

Table 8 shows that the proposed filter achieves smaller SV and closer average positions for each test point than the mean filter. The positioning stability is 6.45, 5.82, and 1.08-fold better for test points (1,1), (1,3), and (3,3), respectively, for the proposed filter compared with the mean filter. The overall average stability is improved 4.45-fold.

In summary, the proposed filter achieved a 20.5% improvement in positioning accuracy, with an overall error of less than 1.46 m and a 65% probability of higher accuracy. Significantly, the proposed filter gained a 4.45-fold improvement in positioning stability.

## 6. Conclusions

Considering RSSI is easily blocked and affected by things and the environment, RSSI-based IPS faces the challenge of an unfortunate application effect. To improve the accuracy and stability, particularly for trilateration-based positioning, we propose a tri-partition RSSI classification as the decreased RSSIs, normal RSSIs, and increased RSSIs, and proposed a novel RSSI tracing algorithm based on k-means clustering as an RSSI filter.

The proposed filter adapts to the real-time IPS, for it shows a characteristic achieving a lower variance (<1 dBm) when the minimum sample size is greater than 20. In contrast, traditional RSSI variation can exceed 10 dBm [26]. The proposed filter offers several advantages compared to the mean filter, including lower variance range and sample variance, and 65% probability to mitigate RSSI left-skewness. Thus, the proposed filter is feasible in real-time positioning.

We design a trilateration-based positioning test within a room. The RSSI propagation model fitting achieves a difference path-loss exponent where 4.613 by the proposed filter while 2.326 by the mean filter. Based on this, the positioning results confirm a 20.5% improvement in positioning accuracy and 4.45-fold improvement in stability for the proposed filter compared to the mean filter. Thus, the proposed filter significantly outperforms traditional mean filtering, providing an excellent option for large-scale IPS improvement.

## Figures and Tables

**Table 1:** Low deviation feature of each sample group compared to sample ALL.

Sample Type	Max Positive Deviation (dBm)	Max Negative Deviation (dBm)	Mean Absolute Deviation (dBm)
Sample 10	1.99	2.51	1.44
Sample 20	2.22	1.572	0.89
Sample 30	1.36	1.17	0.83
Sample 40	1.64	0.87	0.70
Sample 50	1.53	1.56	0.92
Sample 60	1.36	1.17	0.83

**Table 2:** Items count and rate statistics for sample groups. DC, NC, IC represnts the RSSI count of the decreased, normal, and increased collection.

Sample Type	Items Count	DC Rate	NC Rate	IC Rate
Sample 10	103	28%	32%	38%
Sample 20	201	21%	60%	19%
Sample 30	300	26%	51%	23%
Sample 40	400	25%	55%	20%
Sample 50	500	25%	50%	25%
Sample 60	600	19%	57%	23%

**Table 3:** Comparison between the mean and proposed filters in terms of variance range and sample variance over time. SV = sample variance.

Sample Type	Mean Filter				Proposed Filter			
	Max Range	**Min** Range	Average Range	**SV**	Max Range	**Min** Range	Average Range	**SV**
Sample 10	3.2	1.0	1.96	2.28	3.9	1.96	2.25	2.68
Sample 20	3.46	0.15	1.69	2.28	3.08	0.03	1.11	1.54
Sample 30	2.23	0.06	0.66	1.03	1.77	0.01	0.69	0.95
Sample 40	1.62	0.1	0.76	0.96	1.32	0.07	0.67	0.82
Sample 50	1.96	0.3	0.82	1.03	1.25	0.01	0.65	0.83
Sample 60	5.35	0.71	2.12	2.77	2.53	0.13	1.12	1.44

**Table 4:** Skewness reductions by comparisons among filters.

Sample Type	Mitigating	Others Less 1 dbm	Others Less0.5 dbm
Sample 10	70%	100%	66.7%
Sample 20	50%	100%	80%
Sample 30	70%	100%	100%
Sample 40	70%	100%	66.7%
Sample 50	80%	100%	100%
Sample 60	50%	80%	60%

**Table 5:** The simplified RSSI propagation model fitting.

Filter	Filtered RSSI at 1 m (*α*)	Fitted Path-Loss Parameter (*β*)
The proposed filter	–80.14 dBm	4.613
The mean filter	–83.82 dBm	2.326

**Table 6:** Positioning results coordinates at point (1,1), point (1,3) and point (3,1).

Test Point	Positioning Results Using the Mean Filter		Positioning Results Using the Proposed Filter	
	Coordinate x	Coordinate y	Coordinate x	Coordinate y
(1,1)	2.82	1.02	2.69	1.79
	2.56	0.77	2.56	1.63
	4.38	2.83	2.51	1.66
	2.63	0.87	2.74	1.82
(1,3)	2.84	2.25	2.87	2.25
	4.19	2.46	2.97	2.25
	2.80	2.21	2.89	2.27
	4.38	2.10	3.06	2.09
(3,1)	2.48	0.80	2.56	1.18
	2.54	0.51	2.55	1.15
	2.54	0.56	2.54	1.14
	2.42	1.01	2.49	1.22

**Table 7:** Positioning errors and average accuracy promotion.

Test Point	Positioning Errors Using the Mean Filter			Positioning Errors Using the Proposed Filter			Average Accuracy Promotion
	Maximum	Minimum	Average	Maximum	Minimum	Average	
(1,1)	3.84	1.57	2.22	1.92	1.64	1.78	19.8%
(1,3)	3.5	1.97	2.68	2.25	2.01	2.1	21.6%
(3,1)	0.67	0.56	0.61	0.55	0.48	0.51	16.0%

**Table 8:** Positioning stability and increase for the proposed filter vs. the mean filter. SSD represents the sample stand deviation and calculated by the Formula (5).

Test Point	Positioning Stability Using the Mean Filter			Positioning Stability Using the Proposed Filter			SSD Promote
	*STDEV*(*R*)	*AVERAGE*(*R*)	*SSD*	*STDEV*(*R*)	*AVERAGE*(*R*)	*SSD*	(–fold)
(1,1)	1.089	2.215	0.491	0.136	1.775	0.077	6.45
(1,3)	0.81	2.675	0.303	0.109	2.1	0.052	5.82
(3,1)	0.05	0.61	0.081	0.037	0.5	0.05	1.08
